# Seropositive Myasthenia Gravis Triggered by Lyme Disease: A Case Study of Molecular Mimicry

**DOI:** 10.7759/cureus.99366

**Published:** 2025-12-16

**Authors:** Sahil Sardana, Emily Grieco, Samantha MacGavin, Hassan Abdullah Shakeel, Meghan Piccinnin, Taranjit Singh Gill

**Affiliations:** 1 Neurology, Allegheny Health Network, Pittsburgh, USA; 2 Neurology, Drexel College of Medicine, Pittsburgh, USA; 3 Medicine, Allegheny Health Network, Pittsburgh, USA

**Keywords:** borrelia burgdorferi, immune-related myasthenia gravis, lyme neuroborreliosis, lyme's disease, molecular mimicry, myasthenia gravis, neuroborreliosis, nicotinic/acetylcholine, receptors

## Abstract

Lyme disease, caused by Borrelia burgdorferi, is a multisystem infection that rarely produces neuromuscular complications beyond classic neuroborreliosis. Myasthenia gravis (MG) is an autoimmune disorder characterized by fatigable weakness due to acetylcholine receptor (AChR) antibodies. We describe a 74-year-old man with coronary artery disease, chronic kidney disease, and prostate cancer who developed progressive dysphagia, dysphonia, ptosis, and neck weakness. Initial attribution to medication-related angioedema delayed recognition. Neurological examination revealed bulbar weakness and fatigable ptosis. Electrodiagnostic testing confirmed post-synaptic neuromuscular junction dysfunction, and AChR antibody assays were strongly positive across binding, blocking, and modulating subtypes. Concurrent Lyme serologies were positive for immunoglobulin G and immunoglobulin M. The patient required intensive care monitoring and was treated with plasma exchange, intravenous ceftriaxone, prednisone, and pyridostigmine, with marked improvement. This case illustrates a rare coexistence of neuroborreliosis and seropositive MG, highlighting potential molecular mimicry between Borrelia antigens and AChR epitopes.

## Introduction

Lyme disease is a multisystem infectious illness caused by Borrelia burgdorferi, transmitted through the bite of Ixodes scapularis or Ixodes pacificus ticks [1]. From 2010 to 2018, the United States reported an estimated average of 476,000 new cases of Lyme disease annually [2]. The disease is most prevalent in the Northeast, mid-Atlantic, and upper Midwest regions, with 14 states, including Pennsylvania, accounting for 95.2% of all reported cases between 2008 and 2015 [3]. Lyme neuroborreliosis generally develops within 2-3 weeks of infection and has been reported in up to 12% of Lyme disease cases [4]. Although neurological complications of Lyme disease are well-documented, the occurrence of myasthenia gravis (MG) in this context is exceedingly rare. MG is an autoimmune neuromuscular disorder characterized by antibody-mediated disruption of acetylcholine receptor (AChR) signaling at the motor endplate, leading to fluctuating skeletal muscle weakness [5]. In 2021, there were an estimated 17,417 new cases of MG in the United States [6]. We present a rare case of seropositive MG occurring in the setting of Lyme seropositivity, highlighting the potential role of infection-related immune activation in new-onset myasthenic gravis.

## Case presentation

**Figure 1:**
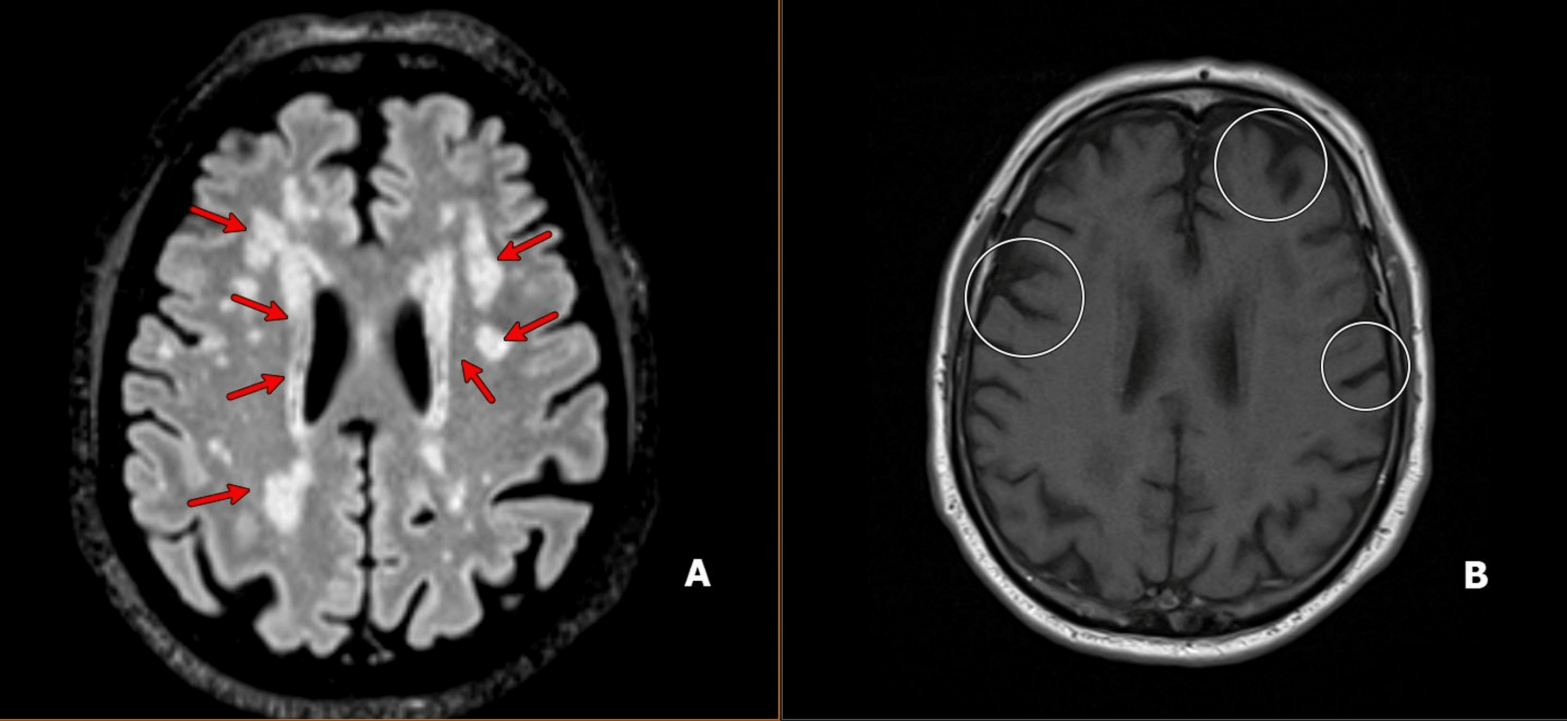
A: Axial FLAIR MRI illustrating chronic small-vessel ischemic changes (red arrows). B: Axial T1-weighted MRI demonstrating mild generalized cerebral volume loss (circles).

A 74-year-old man with a complex medical history, including coronary artery disease, hypertension, hyperlipidemia, chronic kidney disease, and prostate cancer, presented for neurological evaluation of two weeks of insidiously progressive dysphagia and dysphonia following a referral from ENT (otolaryngology). Initial symptoms were attributed to angioedema, possibly secondary to amlodipine, given the temporal association with medication use; however, symptoms recurred despite discontinuation of amlodipine, dose adjustments of his antihypertensive regimen, and corticosteroid therapy. ENT evaluation, including flexible nasolaryngoscopy, suggested possible bulbar weakness during eating. Neurological examination revealed classic bulbar findings, including fatigable ptosis, nasal speech, tongue fasciculations, facial diplegia, and weakness of both neck flexor and extensor muscles. MRI of the brain showed generalized cerebral atrophy and small vessel ischemic changes without evidence of acute infarction (Figure 1).

CT imaging of the chest, abdomen, and pelvis was unremarkable. Due to worsening bulbar symptoms and declining serial measurements of negative inspiratory force and vital capacity, with a nadir of -15 cm H₂O and 0.88 L, respectively, the patient was transferred to the ICU for close respiratory monitoring. Electromyography (EMG) demonstrated post-synaptic neuromuscular junction dysfunction consistent with MG, confirmed by a positive decremental response on repetitive nerve stimulation (Table 1).

**Table 1:** Repetitive nerve stimulation (3 Hz) of the right nasalis (facial nerve) and right abductor digiti minimi (ulnar nerve). The nasalis demonstrates post-exercise facilitation followed by a decremental response, while the abductor digiti minimi shows stable responses without significant decrement. Ampl.: amplitude; mV: millivolts; Ampl 4–1 %: percent amplitude change between 4th and 1st stimuli; Fac Ampl %: facilitation amplitude percentage; Area (mV·ms): CMAP area; Area 4–1 %: percent area change between the 4th and 1st stimuli; Fac Area %: facilitation area percentage; Rate (Hz): stimulation frequency; Train: stimulus train; Time: measurement timestamp; Anatomy: muscle or nerve tested.

Anatomy/Train	Rate Hz	Ampl. mV	Ampl 4-1 %	Fac Ampl %	Area mVms	Area 4-1 %	Fac Area %	Time
R Nasalis - (Facial)								
Setup	1	0.9	20.6	100	2.5	25.8	100	0:00:00
Baseline @ 3Hz	3	0.9	19.1	104	2.6	28.6	103	0:00:17
Post exercise @ 00:00	3	0.9	12.7	99.8	3.5	19	141	0:01:29
@ 0:30	3	0.8	10.1	94.8	3.1	25.8	123	0:02:00
@ 1:00	3	0.8	29.5	88.7	3.0	36.4	119	0:02:29
@ 2:00	3	0.8	32.8	89.1	2.9	49.7	117	0:03:32
@ 3:00	3	0.9	24.7	96.8	2.7	8.1	110	0:04:30
@ 4:00	3	0.7	19.8	79.1	2.5	25.2	99.6	0:05:31
R Abductor digiti minimi - (Ulnar)								
Setup	1	8.3	-0.4	100	32.7	-0.9	100	0:00:00
Baseline @ 3 Hz	3	8.2	-1.9	99.4	32.5	1.2	99.3	0:00:07
@ 0:30	3	7.9	-3.6	96.1	32.5	4.2	99.2	0:02:00
@ 1:00	3	8.9	1.1	108	36.3	6.9	111	0:02:11
@ 2:00	3	9.0	1.7	109	36.0	6.3	110	0:02:20
@ 3:00	3	8.8	0.8	107	35.9	7.4	110	0:02:36
@ 4:00	3	8.3	-1.6	100	34.4	5.5	105	0:03:03

There was no evidence of motor neuron disease on needle EMG. AChR antibodies were positive, including binding (10.50 nmol/L), blocking (71%), and modulating (47%) antibodies, while muscle-specific tyrosine kinase antibodies were negative. Lyme serology returned positive for both IgG and IgM. While Lyme neuroborreliosis can rarely cause bulbar weakness or mimic MG, the clinical picture strongly supported seropositive MG as the primary etiology. The patient underwent five sessions of plasma exchange (PLEX), received a 14-day course of intravenous ceftriaxone for presumed Lyme disease, and was initiated on pyridostigmine and prednisone 60 mg daily for management. He demonstrated a robust clinical response to therapy, returned to neurological baseline, and was discharged home with home care services. At discharge, he was continued on prednisone 60 mg daily and pyridostigmine 30 mg three times daily. The ceftriaxone course was completed via home infusion. The patient was evaluated in the neurology clinic nine days after discharge. He reported no recurrence of symptoms. Prednisone was continued at 60 mg daily, and a follow-up evaluation was scheduled in six weeks.

## Discussion

MG is an autoimmune disorder characterized by impaired neuromuscular transmission, resulting in fatigable weakness of skeletal muscles. The pathogenesis of MG is widely believed to involve immune responses against microbial antigens that share structural homology with epitopes of the AChR, a process known as molecular mimicry [7]. Our patient presented with classic features of MG, including fatigable bulbar weakness, which was confirmed both clinically and via electromyographic studies. The presence of AChR antibodies along with positive Lyme serology (IgG and IgM) supports our hypothesis that Borrelia burgdorferi infection may have triggered the autoimmune cascade through molecular mimicry.

Although Lyme disease was not identified among the infectious agents linked to MG in the systematic review by Leopardi et al., which documented associations with 21 pathogens, most notably Epstein-Barr virus, human papillomavirus (HPV), and poliovirus [8], our case adds to a small but growing body of literature suggesting a potential connection between Lyme disease and MG. Notably, Finsterer (2007) reported a case of a 29-year-old male patient who developed MG with AChR antibody titers nearly 1000 times the upper limit of normal following neuroborreliosis. Despite partial symptom resolution with ceftriaxone, antibody levels remained persistently elevated [9]. Similarly, Evlice and Koç (2018) described a woman who developed seropositive MG two years after successful antibiotic treatment for neuroborreliosis [10]. These observations support the role of Borrelia-driven molecular mimicry in MG pathogenesis. Borrelia burgdorferi has been implicated in other autoimmune sequelae, including neurological complications and Lyme arthritis, through similar mechanisms [11-13].

In our case, the abrupt onset of a severe myasthenic crisis suggested an immunologically mediated trigger. Although 85% of MG patients are seropositive for AChR antibodies, only 17.4% exhibit the full complement of binding, blocking, and modulating antibodies, a profile linked to more severe disease phenotypes [14]. The co-expression of multiple antibody subtypes likely results in synergistic disruption across receptor subunits, thereby exacerbating neuromuscular impairment [15,16]. Vaccination has also been recognized as a potential trigger for MG. Several vaccines, including influenza, hepatitis B, HPV, and most recently, SARS-CoV-2, have been implicated in the onset of MG, often presenting with ocular, bulbar, or facial weakness that improves with acetylcholinesterase inhibitors and immunomodulatory therapies [17-20]. Whether incited by infection or immunization, these cases reinforce the theory of immune-mediated initiation via molecular mimicry.

According to the Centers for Disease Control and Prevention, approximately 9% of patients with confirmed Lyme disease develop facial nerve palsy [21], a manifestation that may closely resemble the facial weakness seen in MG. In our patient, despite serologic confirmation of Lyme disease, an MG workup was promptly pursued given the severity and distribution of bulbar symptoms, ultimately enabling timely plasmapheresis that led to significant clinical improvement [22]. Other infectious agents, including herpes simplex virus and Bacillus Calmette-Guérin (BCG) vaccination, have also been reported in association with MG (Table 2) [23,24].

**Table 2:** Infectious agents and vaccines reported to trigger myasthenia gravis. CMV: Cytomegalovirus; EBV: Epstein–Barr virus; VZV: Varicella–zoster virus; HIV: Human immunodeficiency virus; HPV: Human papillomavirus; HSV: Herpes simplex virus; HTLV: Human T-lymphotropic virus.

Authors	Infectious agent(s) / Vaccine(s) reported
Deitiker, Ashizawa, Atassi [7]	S. cerevisiae, V. cholerae, S. marcescens, E. coli, M. pneumoniae, B. subtilis, S. typhimurium, C. tropicalis, Human herpesvirus 1, Human rotavirus, Hepatitis C virus, Newcastle disease virus, Porcine transmissible gastroenteritis virus, Venezuelan equine encephalitis virus, Vaccinia virus
Wang, Xiang, He, Wang [17]	Influenza virus vaccine
Bahri, Louzir, Othmani, et al. [18]	Hepatitis B virus vaccine
Chung, Lee, Shin, Kang [19]	Human papillomavirus vaccine
Ramdas, Hum, Price, et al. [20]	SARS-CoV-2 vaccine
Schwimmbeck, Dyrberg, Drachman, Oldstone [23]	Herpes simplex virus
Takizawa, Kojima, Suzuki, et al. [24]	Bacillus Calmette–Guérin (BCG) vaccine
Leopardi, Chang, Pham, et al. [8]	CMV, EBV, H1N1 influenza A, Hepatitis B, Hepatitis C, Hepatitis E, VZV, HIV, HPV, Parvovirus B19, Polyomavirus 7, HTLV-1, HTLV-3, Leptospira interrogans, Measles virus, Merkel cell polyomavirus, Poliovirus, West Nile virus, Zika virus, HSV

Multiple pathogens and vaccines have been implicated in the induction of MG through molecular mimicry mechanisms [8,17-24]. Although not previously included in systematic reviews, Lyme disease should be considered a potential trigger for MG, particularly in patients presenting with overlapping signs such as facial weakness. Our case, along with prior reports, supports the need for further investigation into the immunopathogenic role of Borrelia burgdorferi in MG development.

## Conclusions

This case illustrates an unusual concurrence of seropositive MG and Lyme seropositivity, prompting consideration of how infection-related immune activation may shape autoimmune neuromuscular disease. The coexistence of AChR antibodies with recent immune stimulation raises the possibility that molecular mimicry could contribute to the breakdown of self-tolerance at the neuromuscular junction. Although no causal relationship can be drawn from a single presentation, this scenario highlights how immune cross-reactivity may influence symptom onset in susceptible individuals. Further work exploring the interface between infectious exposures and autoimmunity may help clarify the mechanisms underlying such presentations.
